# *Catch the Open!* A Gamified Interactive Immersion Into Open Educational Practices for Higher Education Educators

**DOI:** 10.3389/fpsyg.2022.812091

**Published:** 2022-06-24

**Authors:** Natalia Padilla-Zea, Daniel Burgos, Alicia García-Holgado, Francisco José García-Peñalvo, Mélanie Pauline Harquevaux, Colin de-la-Higuera, James Brunton, Ahmed Tlili

**Affiliations:** ^1^Universidad Internacional de la Rioja (UNIR), Research Institute for Innovation and Technology in Education (UNIR iTED), Logroño, Spain; ^2^GRIAL Research Group, Department of Computer Science, Research Institute for Educational Sciences, Universidad de Salamanca, Salamanca, Spain; ^3^LS2N, UMR 6004, Nantes Université, Nantes, France; ^4^School of Psychology, Dublin City University, Dublin, Ireland; ^5^Department of Financial Accounting, University of South Africa, Pretoria, South Africa; ^6^Smart Learning Institute, Beijing Normal University, Beijing, China

**Keywords:** educational games, gamification, open education, open pedagogies, open practices

## Abstract

Open Education (OE) opens up learning opportunities to, potentially, every person in the world. Additionally, it allows teachers, researchers, and practitioners to find, share, reuse, and improve existing resources under a dependable legal framework. Aiming to spread and foster the introduction of open policies in Higher Education (HE) institutions, the gamified interactive learning experience *Catch the Open!* was developed. *Catch the Open!* targets HE educators who wish to learn, or who wish to deepen their existing knowledge, about OE and Open Educational Practices (OEP). Within the gamified learning experience, the user becomes an educator, Alex, the game character, who receives a task from the Rector: to investigate how to best include OE and OEP in teaching and learning practice within the institution. Alex proceeds to explore and gather information in a web-based 2D virtual HE institution where students, colleagues, and guest researchers will help her to develop a comprehensive understanding of OE and the practical application of OEP. The educational content within *Catch the Open!* is underpinned by an OE competences framework for HE educators, developed in a previous phase of the Erasmus+ OpenGame project. In this paper, the design process to link pedagogical and technological approaches, which results in the *Catch the Open!* gamified web-based interactive application, is presented as well as the application itself. Moreover, two phases of piloting with 153 HE educators from six different HE institutions are presented. The obtained findings showed that the gamified environment helped in learning about OE. On the other hand, learners also suggested several improvement aspects of the gamified environment, such as the length of finishing a learning mission while playing.

## Introduction

“*In building inclusive knowledge societies, Open Educational Resources (OER) can support quality education that is equitable, inclusive, open and participatory as well as enhance academic freedom and professional autonomy of teachers by widening the scope of materials available for teaching and learning*” (United Nations Educational, Scientific and Cultural Organization (UNESCO), 2019).

One aspect of working towards the realization of a more inclusive knowledge society, as envisioned in national and international policy (United Nations Educational, Scientific and Cultural Organization (UNESCO), 2012, 2017, 2019; Dos Santos, 2019) is that of supporting staff in HE institutions to adopt a more open, inclusive educational mindset and to engage in OEP as “the open education movement offers one possible, partial remedy to educational inequality” (Biswas-Diener and Jhangiani, 2017). Miao et al. (2019, p. 9) identify “capacity building in pedagogical use of OER” and “incentives for teachers’ creation and sharing of OER” as two examples of focus areas for OE policy. Where HE educators adopt an attitude or shift their mindset to one of being ready to share their work and/or use the work of others a range of solutions to educational challenges become available to those educators (Inamorato Dos Santos et al., 2017; Nascimbeni et al., 2020).

The educational challenges that can be addressed using an open approach can be at the macro level, such as addressing social justice issues through open education and use of OEP (Hodgkinson-Williams and Trotter, 2018; Bali et al., 2020a,b), for example, providing open education pathways into and through HE for refugees (Brunton et al., 2019; Farrell et al., 2020; Gomez, 2022) or promoting innovation in learning of foreign languages with an open poetry-based approach (Titus, 2017). More micro educational challenges can also be addressed using open approaches, such as reducing costs for students and removing barriers to learning materials by utilizing open textbooks instead of commercial textbooks (Costello et al., 2018), overcoming online social isolation (Huang et al., 2020) or getting students in contact with professional communities by participating in open forums (Morgado et al., 2012). In order to facilitate HE educators engagement with OE and OEP, an ecosystem of enablers is needed, including supporting guides, books, toolkits, courses, (for example, Elder, 2019; Farrell et al., 2020) as well as institutional and national policies.

While several initiatives exist in this context, most of them are not engaging enough (i.e., simple courses and learning materials) or do not connect the used pedagogical approach to teach OER and OEP with real life (i.e., educators cannot see how OER and OEP could be beneficial to them in their real-life). Tlili et al. (2021) highlighted that the rapid evolution of technology is changing OER and OEP design, calling for more innovative approaches to increase users’ engagements with OER and OEP. Motivated by this, and to fill this gap, this study proposes an innovative approach that relies on the benefits of gamification and storytelling in education (Egan, 1989; Tlili et al., 2016) to provide an immersive and engaging teacher experience towards learning the use of OER and OEP. Specifically, this paper presents an immersive and gamified solution to overcome the challenge of encouraging HE educators to engage with OEP.

The Erasmus+ funded OpenGame project (Promoting Open Education through Gamification) contributes to the uptake of OEP and OER use among educators in HE in an innovative and motivating way through a gamified and situated learning experience. The project developed an interactive, online gamified application, *Catch the Open!*, which is based on real-life cases where open teaching approaches have been used by HE educators in their daily teaching practice (Garcia-Holgado et al., 2020). These real-life cases were used to establish a framework with the competences that educators need to develop in order to effectively engage in OEP (Nascimbeni et al., 2020). Using this framework, a comprehensive course curriculum and in-depth content on OEP and OER use were then created, including related reflective activities and self-evaluation tests (de la Higuera et al., 2020), as the base upon which the interactive, online gamified application was designed and developed. Although the link to the project website and the gamified application is provided in this paper, a full reusability of the project’s results can be performed by using the *OpenGame Transferibility Toolkit* accessible at https://opengame-project.eu/wp-content/uploads/2021/10/OpenGame-IO5-Transferability_Toolkit.pdf.

This paper presents an overview of the current state of OE in section “OE and Formative Opportunities for HE Educators: The Current Situation” before presenting a set of related work to highlight the similarities and differences of this project respect to others, in section “Related Works”; section “Catch the Open! The Pedagogical Approach” summarizes the pedagogical approach underpinning the gamified learning experience. *Catch the Open!* is presented in section “Catch The Open! The Technological Approach”. The research method is explained in section “Research Method”, presenting the results in section “Results” and discussion in section “Discussion”. Finally, section “Conclusion and Future Research Directions” outlines the main conclusions and possible future research directions.

## OE and Formative Opportunities for HE Educators: The Current Situation

Stracke (2017) stated that “*Open Education covers and addresses all dimensions related to operational, legal and visionary aspects throughout the analysis, design, realization and evaluation of learning experiences to facilitate high quality education meeting the given situation, needs and objectives*.” However, in general terms, one can say that the main characteristic of OE is its delivery, for free, to anyone *via* Information and Communication Technologies (ICT; Dos Santos et al., 2016b).

OE removes several barriers to involvement in education. OE allows individuals to access learning opportunities or to upskill in a specific field in less expensive and more flexible ways, providing meaningful educational opportunities to diverse cohorts of learners, such as those who are with low financial background. In addition, it can aid in the development of digital competences and offers opportunities for certification at different levels, both formal and informal (Dos Santos et al., 2016a). Furthermore, although HE institutions highlight some barriers to OE (time constraints by academics, or lack of strategy and a shared vision, among others), they also confirmed several motivators from their viewpoint, including higher visibility, students’ access to lifelong learning, free education to all or improving learning outcomes for students, among others (Dos Santos et al., 2016b). Several projects and initiatives in the area of OE have been supported during the last years, such as MOOC-Maker (Alario-Hoyos et al., 2018), OpenUpEd (Rosewell and Jansen, 2014), OPAL, POERUP (Nie, 2013), EMMA, ECO, and MOOQ (Stracke et al., 2017).

This paper is focused on how HE educators can be encouraged to get involved in the OE movement (Ramírez-Montoya, 2015), which enables them to innovate in their teaching and research practices, to create shared-construction experimental laboratories, collaborative academic networks, multidisciplinary projects that transcend contexts, and engage in research with the aim of generating open knowledge (Ramírez-Montoya et al., 2018). Despite this, educators can be resistant to embracing the use of OER and OEP (Dos Santos et al., 2016b). There is a need in HE institutions to motivate educators to utilize open approaches in their teaching and learning practice.

There are several initiatives in this area, such as the Open Education Factory project (Nascimbeni et al., 2019), which proposes several educator-centered proposals to foster the adoption of OEP.

Furthermore, specific courses (OpenLearn, n.d.), web spaces that governments dedicate to OE (Office of Educational Technology, n.d.), and organizations (Scholarly Publishing and Academic Resources Coalition (SPARC), n.d.) provide information on how to start in OE, having a prominent space on OER.

Despite that several initiatives exist in this regard (such as the ones mentioned above), most of them do not provide an interactive and playful experience which are two important features in increasing learning engagement (Wang et al., 2021). Therefore, the OpenGame project draws on several of the initiatives discussed above by offering the sector a set of inspiring practices with the knowledge needed to put them into practice. In addition, it provides the learning content in a story-telling format with a gamified experience in order to foster curiosity and improve motivation of HE educators.

## Related Works

Open Education has been widely promoted to enhance the education openness around the world. The European Commission, *via* its European Commission’s Directorate General for Education, Youth, Sport and Culture, works to promote OE in Europe. Several initiatives have been founded to deepen into this subject. In particular, in relation to this paper, the framework OpenEdu (Dos Santos et al., 2016a) is worth mentioning, which has been taken as a base on which build several studies. One of those initiatives is the *Practical Guidelines on Open Education for Academics* (Inamorato Dos Santos et al., 2017), intended to introduce OE to HE teachers or fostering them to go further, if they already practice OEP. To do so, this guide offers, for each of the 10 dimensions of OE, a list of benefits and challenges as well as a set of reflection questions and recommendations to promote each of those dimensions.

There are other projects intended to promote OE. For example, we can cite the Shout4HE project,^1^ which promotes sharing OEP mediated with technology. In addition, it provides users with a framework to check their advance in terms of OEP and digital skills. Finally, as a result of this project, a set of digital resources, such as videos and eBooks, was also developed and shared.

The project Supporting OER re-use in learning ecosystems’^2^ description indicates that ‘*in higher education to be able to develop a teacher training course, a MOOC available online for the development of competences to adopt OER in an open educational practice (OEP), for a more sustainable option for professionals to develop new skills linked to the uses and development of OER*’. Moreover, one of its results included gamification elements and social networking, but it had to be modified according to the feedback received by the target group, which denied the use of these elements.

Similarly, the OpenMed project^3^ to promote the incorporation of OER and OEP in the countries of the South-Mediterranean. This need arose because there are more students for HE institutions than places and these institutions have to find the way to give everyone the opportunity to access to higher studies. Indeed, this project worked with teachers, institutions, and policy holders in order to provoke a real impact on those countries.

The ENCORE+ project^4^ (Ehlers and Kunze, 2021) maps the most relevant OER in different institutions around Europe and combines the efforts of the academia in producing and maintaining these resources with the needs detected in the working market, trying to merge both views. Moreover, this network also supports the elements needed to boost the OER: technology, policy, quality, and innovation.

As highlighted in this section, a great effort is being done by European Commission to promote OE in HE. Since we can find several related projects, which have a similar aim that OpenGame, we could indicate two major innovations: a comprehensive view of the OE, including information and promotion of OER and OEP; and gamification that aims to motivate users by using different game design elements (e.g., badges) and a narrative to guide the learning process.

## *Catch the Open!* The Pedagogical Approach

*Catch the Open!* is a gamified learning online application aimed to facilitate engagement in a learning process about OE and OEP. Thus, an important element in the development of this application was the underpinning pedagogical design. *Catch the Open!* was developed on the basis of the previous outputs from the OpenGame project, namely: an OE competences framework for HE educators; and a set of 24 real-world practices which, through a careful scrutiny, were established to be at the core of the process (Garcia-Holgado et al., 2020).

In addition, some of the features of the resulting application had to be considered during the design process: the learning will be self-directed and autonomous; many OER already exist in this area; and there are no constraints about what lessons must be taken or in what order they should be taken. With this information, educational content to be included in *Catch the Open!* was designed with the following characteristics: (1) self-contained with extra resources, (2) reusing and remixing existing OER, and (3) no predefined order in contents.

The methodology followed to develop this content was based on three stages: (1) structure definition, (2) practices distribution and adaptation, and (3) knowledge search. Each stage is described below:

### Structure Definition

Within the OE competences framework for HE educators (Garcia-Holgado et al., 2020), eight HE educational challenges that can be addressed by using OEP were identified and then eight competence areas related to those OEP were defined. In the development of the course curriculum and content that underpinned *Catch the Open!*, eight modules were developed that are aligned to the OE competences framework. A module presents the main path of the learning process, based on the educational practices selected in the OpenGame Handbook of Successful Open Practices (Garcia-Holgado et al., 2020). In addition, eight units of learning (UoL) were also defined, to widen the knowledge available within the course curriculum and content about OE. Each module is linked to two UoL based on which competences align with which OEP (see Table 1).

**Table 1:** Module identification, module title, competences, and learning units.

M#	Module title	Competences to be developed	Learning units
A	Use OER in your teaching activities	1. Use open licenses 3. Create, revise and remix OER	1. Use open licenses 3. Create, revise and remix OER
B	Release your teaching resources as OER	1. Use open licenses 2. Search for OER 3. Create, revise and remix OER 4. Share OER	2. Search for OER 4. Share OER
C	Use OER produced by other educators and experts	1. Use open licenses 2. Search for OER 3. Create, revise and remix OER	2. Search for OER 3. Create, revise and remix OER
D	Share lesson plans and content with other educators	5. Design open educational experience	4. Share OER 5. Design open educational experience
E	Use OER to address learners’ preferences and learning needs	1. Use open licenses 5. Design open educational experiences 6. Guide learners to work in the open 7. Teach with OER	5. Design open educational experiences 7. Teach with OER
F	Co-produce your content with your students as OER	1. Use open licenses 6. Guide students to work in the open 7. Teach with OER	1. Use open licenses 7. Teach with OER
G	Open up assessment to real-world contexts	8. Implement open assessment 6. Guide students to learn in the open	8. Implement open assessment 6. Guide students to learn in the open
H	Support students to learn in the open	6. Guide students to learn in the open	6. Guide students to learn in the open 8. Implement open assessment

Scoring: (A) a set of three bars is presented for each of the rooms, with the information about practices (red), units of learning (blue) and tests (yellow); (B) global and local scoring; (C) global scoring and button to access the global and local scoring; and (D) for the current room, a doughnut chart is shown for each of the categories.

Additionally, the course duration was adjustable and is intended to correspond to what a teacher can reach in a (full) day’s work, in a (part-time) week-long effort, or in a course over a semester. More precisely, the course was designed to take 8 hours in the basic version, 16 h in the medium version, and 32 h in the long version.

### Practices Distribution and Adaptation

Practices in the Handbook of Successful Open Practices (Garcia-Holgado et al., 2020) are the base to propose the learning activities. For each module, three practices are included: one of which will be qualified as the ‘main practice’ that is associated with the basic version and is always shown, and two other practices that are used in the longer versions of the course and gamified learning experience.

Learning activities are included in the course with the goal of inspiring learners to introduce OEP into their daily teaching and learning practice. Moreover, besides giving them a summary of a practice, learners are encouraged to reflect on how they could introduce that practice in their regular classes.

### Knowledge Search

An additional constraint was self-imposed: each module and each UoL followed the same outline in order to ease the design of *Catch the Open!* application. This resulted in the modules having a common outline, ensuring a consistent approach in the development of the course curriculum and content:

1. “Welcome to this module”: A short introduction, typically using an inspiring video;
2. “More about open education?”: In the medium and long version, this section provides the learner extra background for the topic of the module;
3. “Discover the practice!”: The practice is at the core of the module and will be the inspiring activity that generates ideas for learners. In this section, a short summary of the practice is included, with an option to explore the topic in more depth information if desired;
4. “What do we need?”: Here, the learner is made aware of what is needed to obtain the competences related to this OEP;
5. “A little bit more about…”: Information is presented here relating to the first linked skill and the user is redirected to the corresponding UoL;
6. “And also about…”: Information is presented here relating to the second linked skill and the user is redirected to the corresponding UoL;
7. “Let the learning activity start!”: The learner is invited to reflect on the content they teach in order to analyze if it is appropriate to apply the practice in that context;
8. “More to explore…”: In the medium and long versions of the course, a set of questions are presented that act to make the learner reflect on how the practice might be implemented in their context;
9. “Some thoughts about it?”: The learner is encouraged to select the option that bests fits their reflections on several questions related to the practice applied to their teaching and learning practice;
10. “Let us discover two other practices!”: In the long version of the course, two other complete practices, including learning activities, are presented;
11. “What have we learnt?”: A summary of the content presented in the module are outlined.
12. “Time to pick up my new badge!”: An adjustable question with several levels of mastery for the related competence is posed to the learner. After a self-reflection process the learner will decide how skilled they are in relation to that competence.

Similarly, all the UoL have a common outline:

1. “Let us do some learning!”: A reading or a video about the content is presented.
2. “To learn a little more…”: In the medium and long versions of the course, a second related resource is presented.
3. “With even more time…”: In the long version, another resource (reading/video) is presented.
4. “Let us conclude”: An activity is presented that relates to the skill taught in the learning unit.
5. “And finally for the badge”: As for modules, a self-assessed question is presented.

With this information, templates for modules and for UoL were developed. In order to establish which sections correspond to each of the knowledge levels (basic, medium, long), a color code was introduced in the template.

Once the templates were provided, the project team developed the content, activities, and quizzes, which would later be distributed as an OER (de la Higuera et al., 2020).

## *Catch the Open!* The Technological Approach

As previously stated, the main result of the OpenGame project is a gamified application to foster participation of HE educators in OEP. This application presents the educational contents explained in the previous section, inserted in a context-sensitive narrative: an educator, Alex, is asked to investigate how to best include OE and OEP with teaching and learning practice in the institution.

### *Catch the Open!* The Technological Design

*Catch the Open!* is a web-based application, available at https://opengame-project.eu/game/. It has been developed by using the PHP framework Laravel and a MySQL database for the backend; and HTML5 and CSS with the framework Angular for the frontend. Additionally, a system to easily present the information has been developed by using json files which contain educational and playable contents in several languages.

Registration is needed to access the application, allowing being able to record several data about learners, as well as information about interaction with the application and learning achievements. Moreover, with the data recorded for each learner, several analyses can be performed, based on gender, age, teaching expertise, etc.

Finally, a system to automatically present the application in the learner’s native tongue is included. Since both the course curriculum and content and *Catch the Open!* itself are available in five languages, a learner can utilize the application in their preferred language. Regardless of the manual option, the language automatically selected will be the one obtained from the default language in the navigator running the application. If that language is not one of the languages available in the application (Spanish, English, French, German, and Portuguese), the application will be presented in English.

### *Catch the Open!* The Gamification Approach

The most used definition of gamification is the use of game design elements in non-game contexts (Deterding et al., 2011). Although the most frequently used gamification approach is based on points, badges, and leaderboards (PBL), Deterding’s classification (cited by Dicheva et al., 2015) includes the following elements: goals/challenges; personalisation; rapid feedback; visible status; unlocking content; freedom of choice; freedom to fail; storyline/new identities; onboarding; time restriction; and social engagement.

Taking into consideration the features of our target group, HE educators, and resources limitations, we eliminated the possibility of multi-user interaction. However, learners have a clear idea about goals since the introduction to the application includes a brief text explanation of the adventure and an initial dialogue presents the main narrative. Regarding personalization, we utilized profile-centered design, considering different initial skill levels and allowing users to choose freely what rooms (modules) to visit or not. Feedback is constantly provided through the use of pop-ups, dialogues, and achievement tracks. Learners should also be familiar with the context of the narrative given it is designed in a HE institution-based scenario, with educators, students, and researchers as characters.

Moreover, in *Catch the Open!* we have included an explorative, context-sensitive world (classes, laboratories, and auditoriums of the institution), an inventory to save items, locked functionalities to be unlocked with items or actions, conversational instructions, and a scoring system that considers both instructional and playable actions. We describe these elements below.

**Figure 1:**
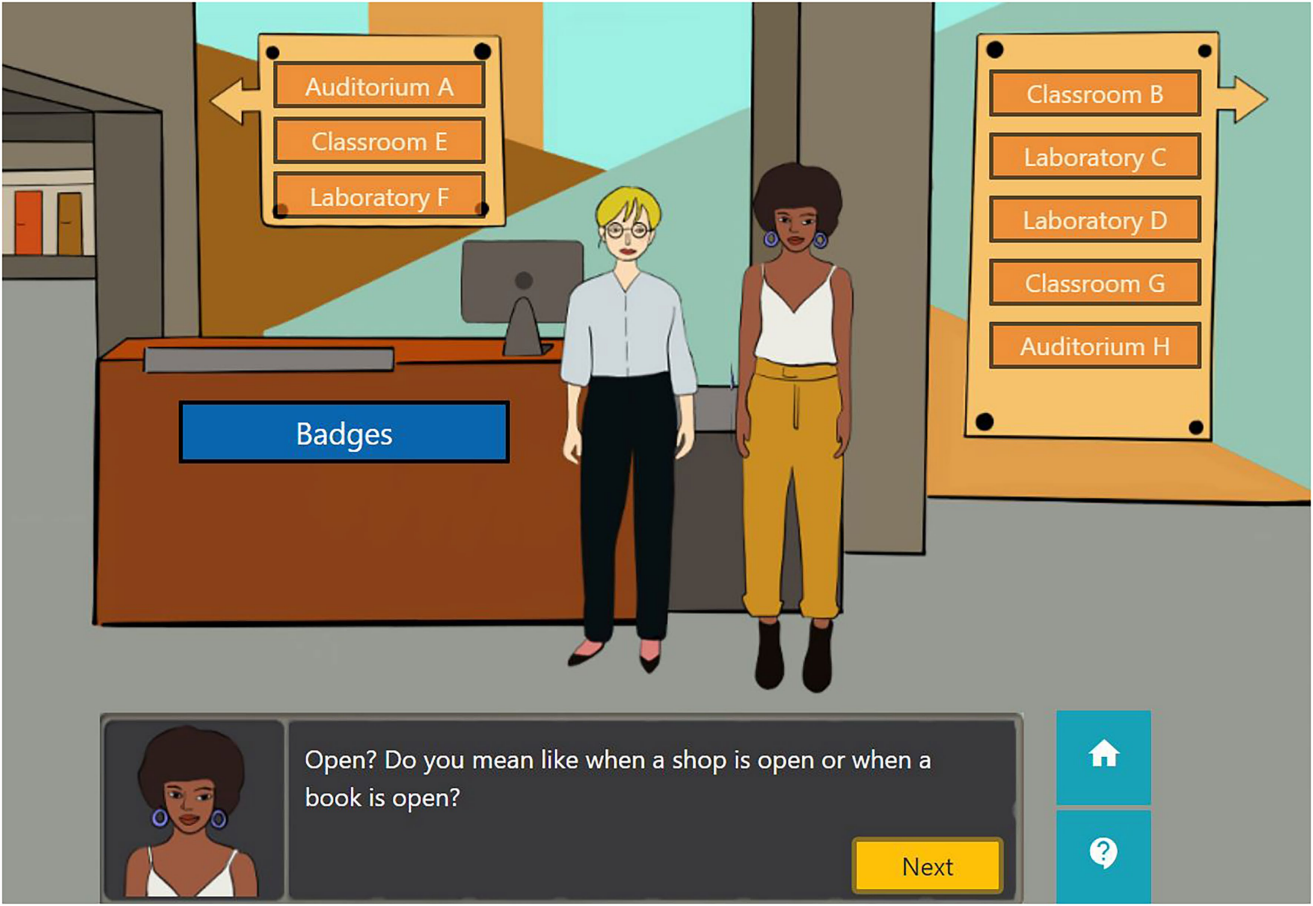
Hall scene.

Learners explore a HE institution-based scenario where they discover the story by reading several dialogues between the protagonist and other characters. There is a main scene from where all other rooms can be reached: it is the building hall, which has several navigation options that lead to classrooms, laboratories and auditoriums (see Figure 1).

**Figure 2:**
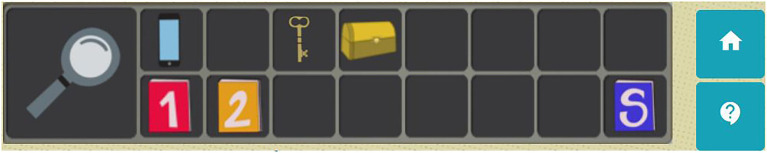
Inventory available in the lowest part of the screen.

When the protagonist comes into a room, she usually finds one or more people and she talks to them. Those conversations allow the user to follow the story, which also presents the educational content in a contextualized way. Additionally, to learn contents in the different levels, the user has to unlock them by finding some items in the scenario or by using a previously obtained item. The items obtained during each of the scenes are shown in the inventory, which are always available at the lowest part of the screen (see Figure 2).

**Figure 3:**
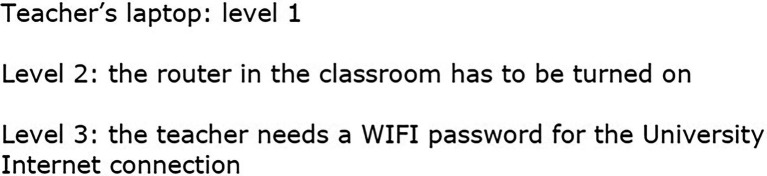
Part of the design documents.

Instructions about what to do in each moment are presented in the dialogues, but users can always click on the “Help” or the “Magnifying” buttons to obtain more information. These instructions are intended to be clear in order to avoid dropouts of learners who are not familiar with game mechanics. An example of the mechanics to pass levels in a scene is shown in Figure 3.

To allow users to keep track of their progress, the scoring is shown in the upper right corner of the screen. Both educational and playable actions increase the scoring and more detailed information can be checked while playing, both for local and global progress, and for each of the educational content, as explained in “Catch the Open! Educational and Playable Monitoring.”

### *Catch the Open!* Link to Pedagogical Approach

The gamification approach is intended to establish a link between the learner and the course by means of a feeling of identification, i.e., real-life scenarios. This fact, together with the intrinsic motivation of gamification, tries to maintain learners’ connection to the process of learning about OE and OEP.

**Figure 4:**
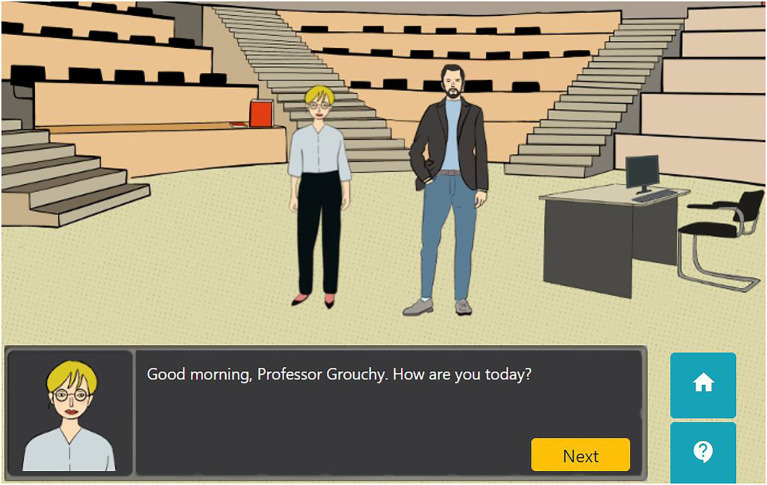
Auditorium A.

**Figure 5:**
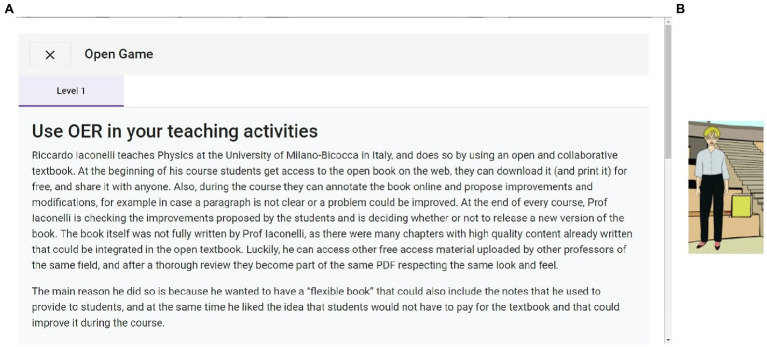
**(A)** Partial view of the open practice presented to the user; **(B)** highlighted book to show where the user has to click on.

This section explains how pedagogical contents are presented during Alex’s adventure. Auditorium A is explained in this section to show how the link between the narrative and the OE content is established. To come into Auditorium A, the learner has to click on the label with that name (see Figure 1). The Auditorium A (see Figure 4) presents a situation where Alex has to face a grouchy teacher who does not like students’ attitudes and has no faith in their future. This teacher does not like Alex’s task because he thinks that using different pedagogies will be a waste of time. However, Alex presents him with one of the open practices to convince him about how it can improve students’ motivation (see Figure 5A). To be able to present the practice, Alex has to click on the book in the Auditorium (see Figure 5B).

**Figure 6:**
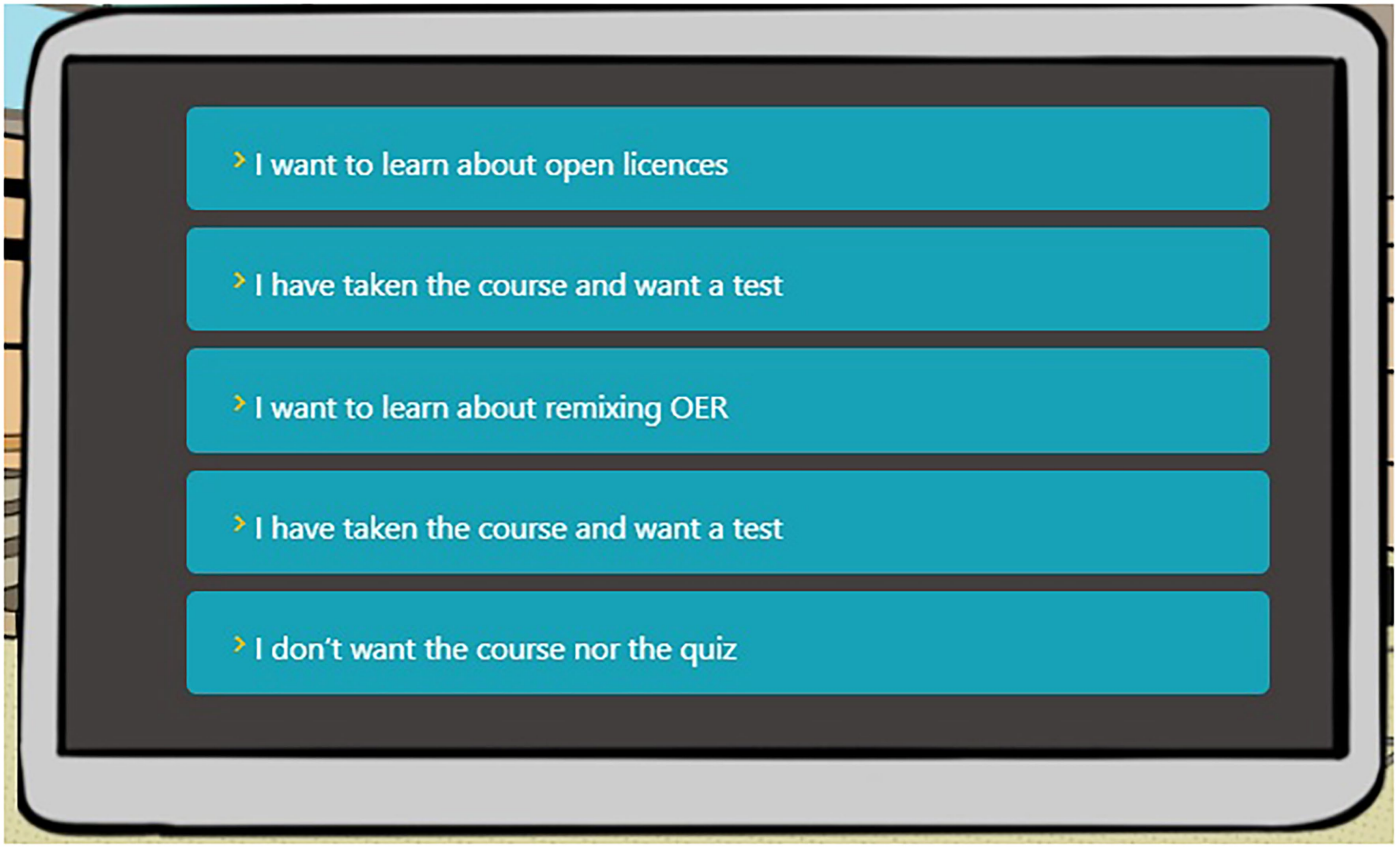
Options to learn or to test the units of learning associated to the current module.

As previously stated, each module has two UoL associated, which explain the theoretical contents included or related to the open practice. For that reason, when the learner finalizes their reading of the text in Figure 5A, options to learn about the associated UoL are presented (see Figure 6). The learner can engage in further exploration about the skills presented in the UoL or attempt the test in either UoL; or the learner can opt to do nothing further and continue on to other modules.

**Figure 7:**
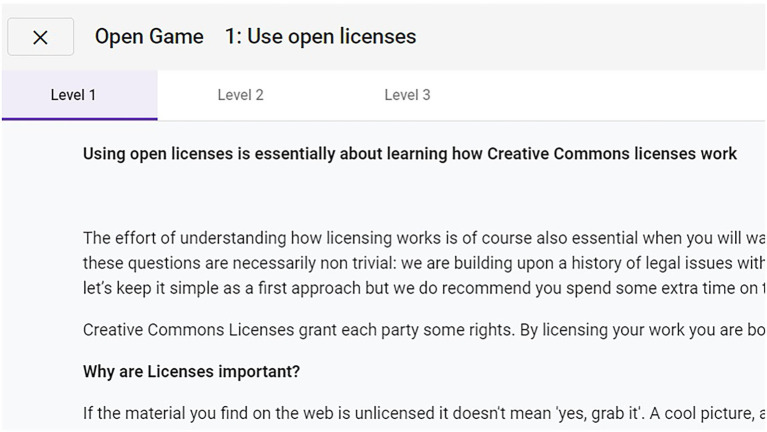
Information of the unit of learning, with the different levels in different tags.

When the learner chooses to visit the content in one of the UoL, the information is displayed in a modal dialogue, offering access to the three levels in which the content is divided (see Figure 7). If the learner opts to take the test, a five-question multiple-choice test is presented, related to the content in the UoL. It is worth mentioning that tests in the course curriculum and content (de la Higuera et al., 2020) have 10 questions; however, an adaptation process was performed in order to avoid demotivation for those long tests at the same time that the knowledge obtained can be assessed.

**Figure 8:**
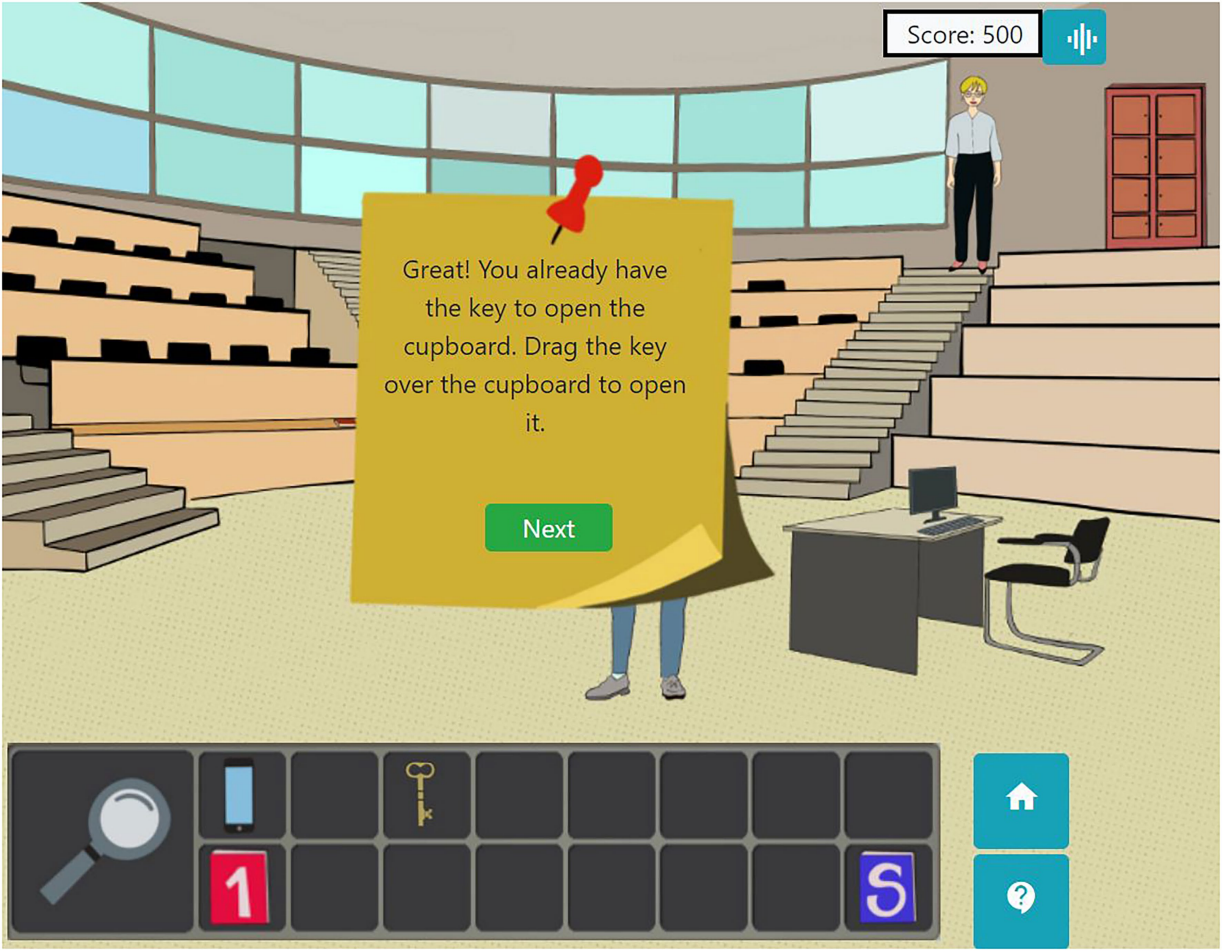
Instructions to access level 2 of Module A in the Auditorium A.

However, levels in modules are managed by means of the story. If the learner decides to continue on with the adventure, levels two and three are reached. If the learner decides to do the self-reflection test, the learning process in that room is defined as being finished. In Auditorium A, to continue to level 2, Alex has to open a cupboard with a key, which Professor Grouchy gives her after a brief conversation. More information (module A, level 2) will be found inside the cupboard (see Figure 8).

**Figure 9:**
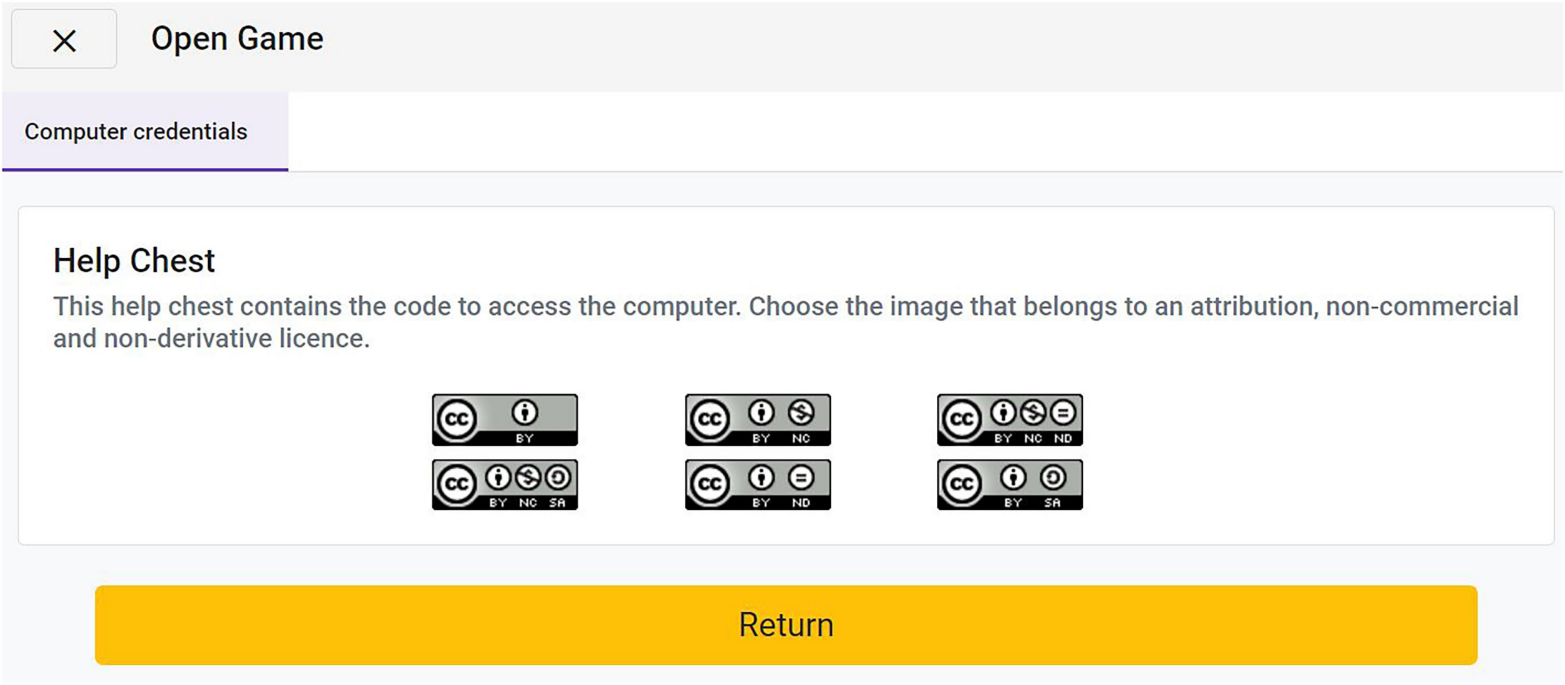
Puzzle to reach level 3 in Module A, in Auditorium A.

If the learner decides to continue exploring, he or she has to reach level three by solving a puzzle, which Professor Grouchy will present to Alex (see Figure 9).

In any case, the learner can re-visit any of the rooms and access the module information by clicking in the numbered book saved in the inventory (see Figure 2).

Finally, the competences trained in the application are highlighted in the “Badges” section (see the option “Badges” in Figure 2). In that section, all the modules together with their related UoL are presented. At the beginning, all of them are shown in grey. As user overcomes the evaluations (tests), UoL titles turn to a green color. If a module has one green UoL, it turns to yellow; if a module has the two UoL in green, it turns to green as well, meaning that the competence linked to that module is already achieved.

### *Catch the Open!* Educational and Playable Monitoring

One of the game mechanics which can be included in a gamified application is the progress perception. In *Catch the Open!* we included a scoring system, which is intended to act as feedback both for educational and playable actions, having a maximum of 12.000 points for each room. Out of these 12.000 points, 8.000 are linked to actions for modules and 4.000 to actions for UoL. There are two special behaviors with the scoring system, namely: (1) the scoring regarding the UoL tests just can be obtained complete or nothing (a UoL is not overcome until the test is fully well answered); and (2) tests regarding modules are fully rewarded independently of the answers provided, since they are related to intentions and thoughts of the respondent: the feedback for each answer is about congratulating the initiative or providing ideas to overcome difficulties.

In *Catch the Open!*, learners obtain a triple-ended information with local and global approaches: in the upper right corner of the screen, the user can see how the scoring increases while he or she visits the rooms (see Figure 8). By clicking on the button next to the scoring, learners can see graphical information about the points obtained for reading the practices, reading the UoL and solving tests, for each of the already visited rooms. Moreover, in a second tap, the user can see a doughnut chart for each of those parts, related to the current room.

**Figure 10:**
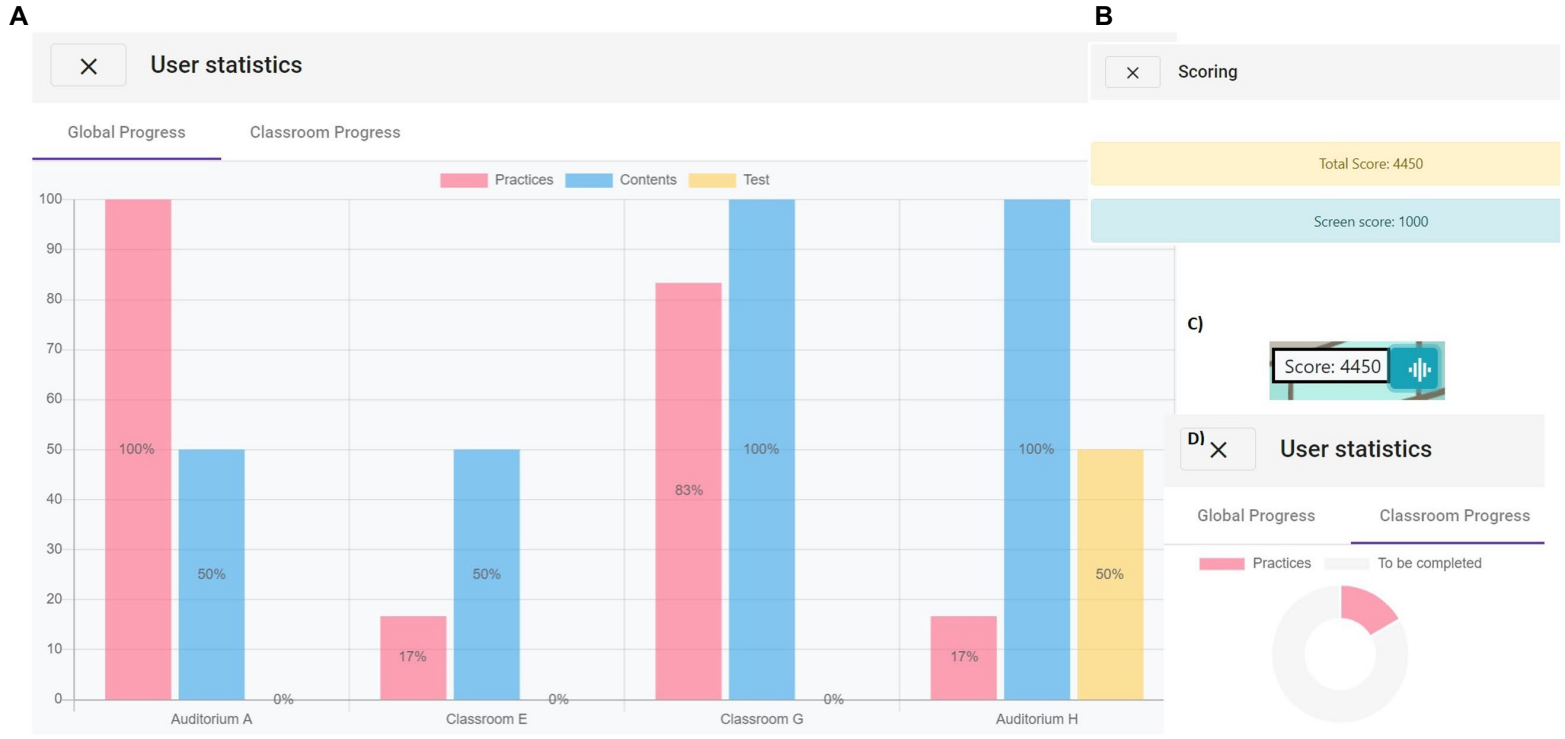
Scoring: **(A)** global scoring and button to access the global and local scoring; **(B)** global and local scoring; **(C)** a set of three bars is presented for each of the room, with the information about practices (red), units of learning (blue) and tests (yellow); and **(D)** for the current room, a doughnut chart is shown for each of the categories.

Finally, by clicking the button “S” (see Figure 8), the user can see the scoring out of the total which has been obtained in the current screen. The set of images about scoring can be seen in Figure 10.

## Research Method

The application development process followed a user-centered approach, involving the users from the very beginning. For this reason, the piloting had an important role, involving different types of users (learners, players) and using different techniques to collect information. Thus, the piloting was divided into two phases: the first phase was conducted with the alpha version, and it comprised two activities: a heuristic evaluation by experts, and an internal alpha-testing by the project partners; the second phase started with the launch of the beta version and continued until the *Catch the Open!* v2.0, covering an internal testing with partners and an external testing with external users. In the following sections we describe each of those phases.

### Phase 1: Alpha Version. Heuristic Evaluation and Internal Testing With Partners

The heuristic evaluation is an inspection technique that aims to identify usability problems. Evaluators review the interface and judge its compliance with recognized usability principles called heuristics. User testing and heuristic evaluation are complementary methods. Meanwhile experts identify more high-level structural problems during a heuristic evaluation, user testing would be able to assess the usability issues most pertinent to users much more directly (Tan et al., 2009).

According to Vieira et al. (2019), there is a lack of heuristic modeling works for educational games. However, there are different authors that have developed or adapted heuristics for game design, such as (Desurvire et al., 2004; Pinelle et al., 2008; Jerzak and Rebelo, 2014). Moreover, there are more specific heuristics for measuring motivational affordances, such as the 28 gamification heuristics developed by (Tondello et al., 2016, 2019). On the other hand, although there is no standard, the Nielsen heuristics (Nielsen, 1994) are the most widely used in web application or mobile apps. In this sense, the Nielsen Norman Group has produced a guide on how to apply them to video games (Joyce, 2019).

In this context, we used the Nielsen heuristics due to the game developed is a web application, so the interaction is quite similar to other web tools. Moreover, during this first phase, we were looking for structural problems not only focused on the gamification, but also in the user interaction.

We involved three experts of different age ranges and all with extensive experience in the world of video games, both in the field of entertainment and educational use. The recruitment of the experts was carried out in the Spanish context, paying special attention to involving experts who had not prior knowledge about the project. The invitation to conduct the heuristic study was made *via* email. The final set of experts was the following: a female software engineer between 20 and 30 years old, a male telecommunication engineer between 30 and 40 years old, and a male software engineer between 40 and 50 years old. All the experts were informed in advance that the game was an alpha version.

The heuristic evaluation was completed with an internal testing with the partners, making a total of 9 testers. A 10-item questionnaire were used to collect the feedback. This user testing did not involve predefined tasks for the users. All items were a 3-points Likert scale (yes, more or less, no):

1. I can distinguish one room from another.
2. I know how to go to other rooms.
3. I know the objects I have collected.
4. I know the objects from my inventory that I can use at every moment.
5. I know how to use elements in the inventory.
6. I know how to use the elements in the scene.
7. Common actions are done as you would do it in a regular video game.
8. I can identify interactable elements.
9. I know what to do in every moment.
10. The information presented in the screen is the one I need in each moment, no more.

### Phase 2: Internal and External User Testing

The second phase was focused on testing the beta version (alpha version enhanced with experts’ feedback) with both internal (partners) and external users, including primary users (higher education teachers), and secondary users (future teachers).

Different approaches were taken to disseminate and test the beta version of the course: embedded in a subject’s Learning Management System (LMS), sharing the link *via* Tweeter, as invited talks in specific courses, or as part of a longer course. Some of those approaches also included live sessions showing how to use the application. In all the cases, partners shared the link to the game and the link to a questionnaire to collect the users’ feedback, including an open-text space to provide recommendations. Since this evaluation process was developed along the production phase, some of the results correspond to versions where some functionalities were still missing, badges were not implemented, and some bugs needed to be fixed.

Regarding the recruitment process for the evaluation, it was not specifically designed for *Catch the Open!*, but on the contrary, our gamified experience was included as a part of specific course being developed for users with profiles in our target group. That way, PhD students evaluated the application because they used it as part of their doctoral courses; HE teachers taking a course of Higher Education Digitalization, received an invited talk and an activity to be performed with *Catch the Open!*; or partners with a high level of expertise in OE and a vast community of followers in the area, launched the proposal *via* Tweeter.

The developed questionnaire is composed of the following six sections:

1. Demographic context. This section includes six items to collect gender, age range, PhD degree, academic position, knowledge area and academic expertise.
2. Application usability. Three 5-point Likert scale items based on the same heuristics used during the phase 1 (Joyce, 2019). Items Q7-Q9 in Table 2.
3. Gamification strategy. Three 5-point Likert scale items based on (Toda et al., 2019). Items Q10-Q12 in Table 2.
4. Contents. Four 5-point Likert scale items to collect feedback about the appropriateness of the content delivered during the learning experience. Items Q13-Q16 in Table 2.
5. Overall opinion. Three 5-point Likert scale items to get general opinion about the experience with *Catch the Open!* Items Q17-Q19 in Table 2.
6. Viewpoint. A final open question to highlight positive and negative aspects.

**Table 2:** User evaluation items.

**ID**	**Item**
Q7	I know what to do in every moment
Q8	I know how to use all the elements
Q9	I know how to move to every room
Q10	The metaphor used in the application is attractive in this context
Q11	I obtain rewards when my performance is correct
Q12	I understand the goal I have to achieve in the metaphor
Q13	The contents provide proper information about Open Education
Q14	The content extension is appropriate to the time allowed
Q15	The feedback from quizzes is clear and understandable
Q16	Questions in quizzes are related to the content associated to the scene where they are presented
Q17	I liked the application
Q18	I learnt about Open Education
Q19	I find the information in the game useful for my daily teaching

A total number of 62 users were registered in the beta version of the application. Although an explicit identification of the country was not collected, we can analyze the users’ origin from their reported home institutions: 41 from Spain, 8 from Germany, 6 from France, 2 from Portugal, 1 from Ireland, 1 from Colombia and 3 not known.

A set of bugs and recommendations were compiled from the beta version, which were analyzed by the development team together with the rest of the consortium. Once categorized, new features were included in *Catch the Open!* which would be the v1.0. This version was released on 2021 June, the 4th, having a total number of 131 users between June and November. Different on-the-fly updates were performed in this time, obtaining a v2.0, including: more information in the registration form, graphics about scoring, translations into some languages, and help screens.

Moreover, two additional pilots were conducted in two different online courses at the universities of Palestine (An-Najah National University) and Colombia (Universidad Nacional de Colombia) in December 2021. A total number of 80 users participated in these pilots, 59 out of which filled in the questionnaire.

The final sample for the test in this second phase was as follows. A total number of 74 individuals whom profiles are: 41 males, 22 females, and 11 unknowns. The age ranges distributed with 21 participants between 20 and 30, 27 between 31 and 40, and 26 between 41 and 50. In addition, 22 of the participants had a PhD degree and 30 were PhD candidates. Among the 74 respondents, there were 4 full professors, 11 Assistant professor /Lecturer, 16 Assistant lecturer / demonstrator /seminar leader /associate lecturer/graduate teaching assistant/departmental lecturer, and 21 researchers; 22 of them did not record their position. Regarding their research or teaching area, we have 20 participants from Technology, 33 from Education, 8 from Social Sciences, 12 from Educational Technology and 1 from Communication Science. Their academic expertise is mainly concentrated in the range 1–5 years (32), but other participants have a background of less than 1 year (7), between 6 and 10 (18), between 11 and 15 (13), and more than 15 years (4). However, for the statistical analysis, we split the experience in novel (until 5 years) and experts (more than 5 years).

## Results

**Figure 11:**
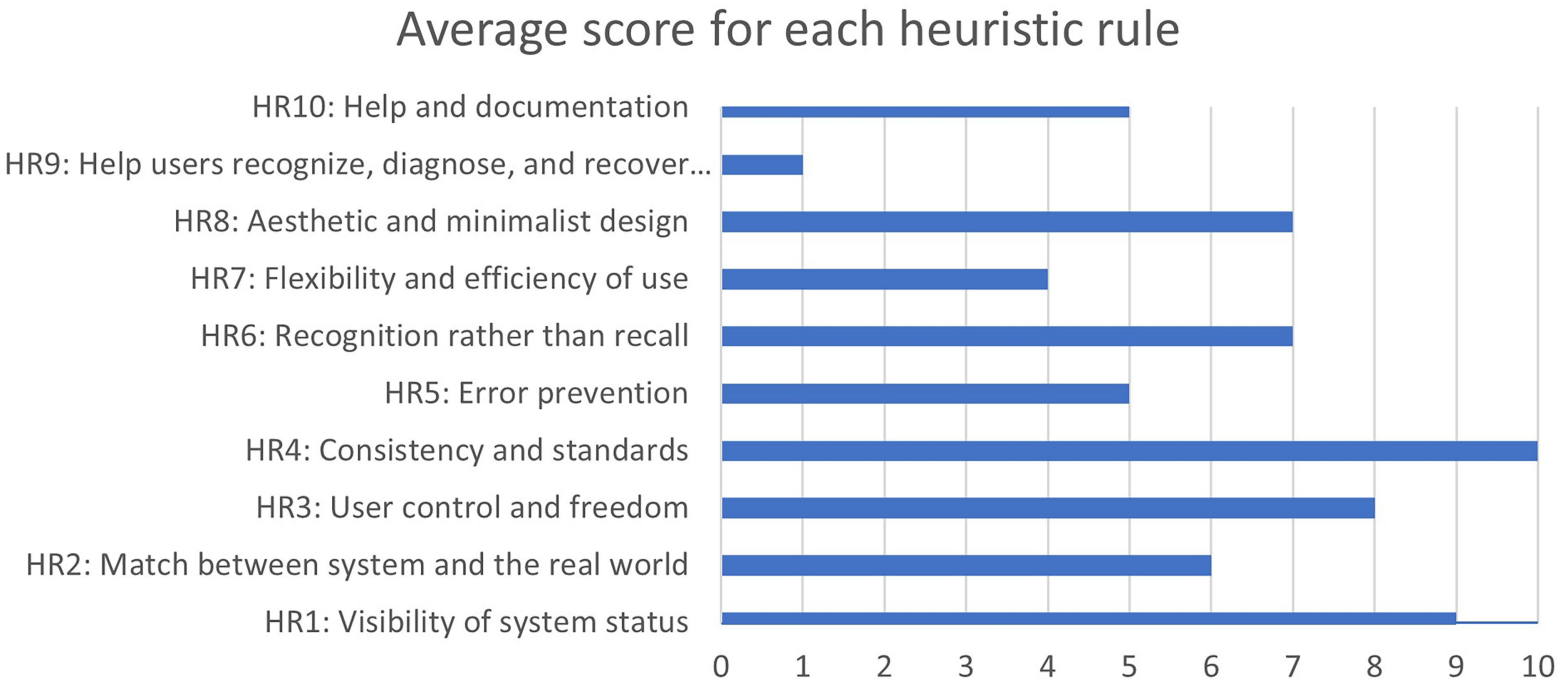
Final value for each heuristic rule.

In the first phase, a heuristic evaluation was performed. The three experts involved in this task detected problems associated with all heuristic rules (Figure 11). The heuristics that present the worst value are Visibility of system status (HR1), User control and freedom (HR3) and Consistency and standards (HR4).

The evaluation identified major problems that were solved in the beta version, such as “The help button takes you back to the start screen if you are in the hall” (HR10) or “There is no freedom in the game, you only have one path to follow” (HR7). On the other hand, regarding consistency, the main problems detected were the lack of interaction with the items, which was not a problem of the game but a missing feature in the alpha version. Moreover, experts indicated that “The aesthetics are out of date” (HR9); however, the visual design follows a vintage style.

**Figure 12:**
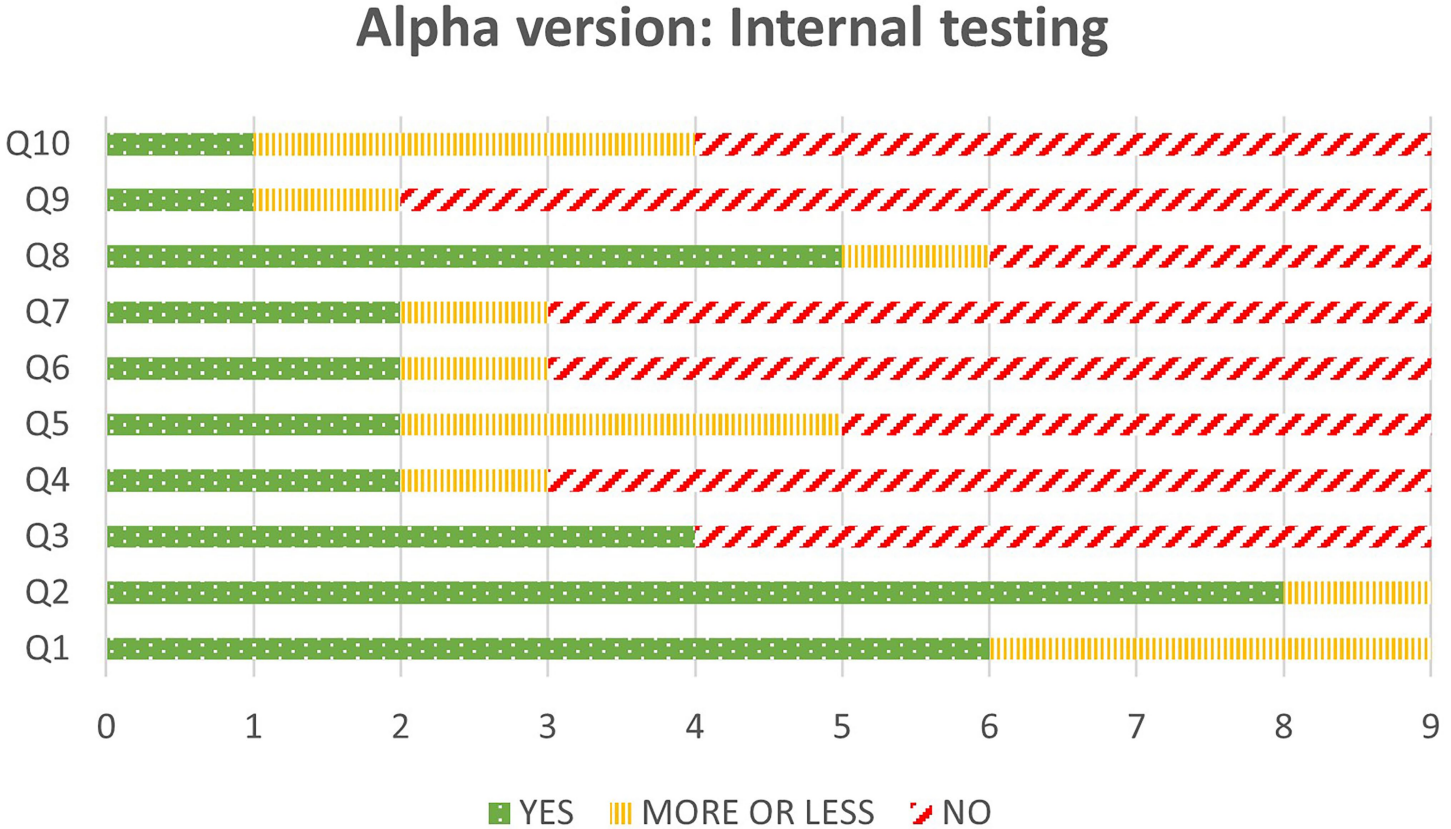
Final value for questions in the internal testing.

To complete this heuristic evaluation, the results obtained from the nine partners are shown in Figure 12, where we can see how the lowest valued questions are Q4, Q6, Q7 and Q9, meaning knowing the objects from the inventory that can be used at each moment, knowing how to use the elements in the scene, working as a regular video game, and knowing what to do in each moment.

Furthermore, some key explanations from the testers, even in other questions, showed us how to solve some of the main problems that they found (just a brief sample is selected):

*… nothing allows me to know what distinguishes a classroom from a laboratory (tester 4).*

*… the university map is not very useful because there are no indications of places and rooms (tester 3).*

*… I have trouble finding my way through the phases of the game, to identify what objects should be collected, for what purposes? (tester 2).*

*… sometimes it is not yet very clear when I can use them (the objects in the inventory) for example (tester 3).*

*… Except when the text guides, the use of the objects remains difficult to understand… (tester 2).*

*… Sometimes I do not know what to do, there are the L1, L2, L3 which are displayed and I understood that this corresponded to the 3 levels of lessons but to someone new to the game it’s not that clear … (tester 3).*

*… I read the module and then nothing happens I can just close the box (tester 3).*

*… There are words like “true,” “false,” “correct,” “no.” I would suggest a green red code to understand when we have the right answer and when we do not… (tester 4).*

*… Classroom B - ‘possible options to publish’ activity - I am unsure if this activity is not working or if I just do not know what to do … (tester 6).*

*… Their function (objects in the inventory) is not clear to me but it is clear that I can click on them at any time (tester 7).*

*… I got L1, L2 and L3 without knowing where they came from … (tester 8).*

*… The confusing part is having actual objects mixed with abstract items called L1, L2… (tester 9).*

Since it was an incomplete version, all the comments were considered to re-design some elements, to include some help tips when needed and to fix some bugs which were found in this test phase.

**Figure 13:**
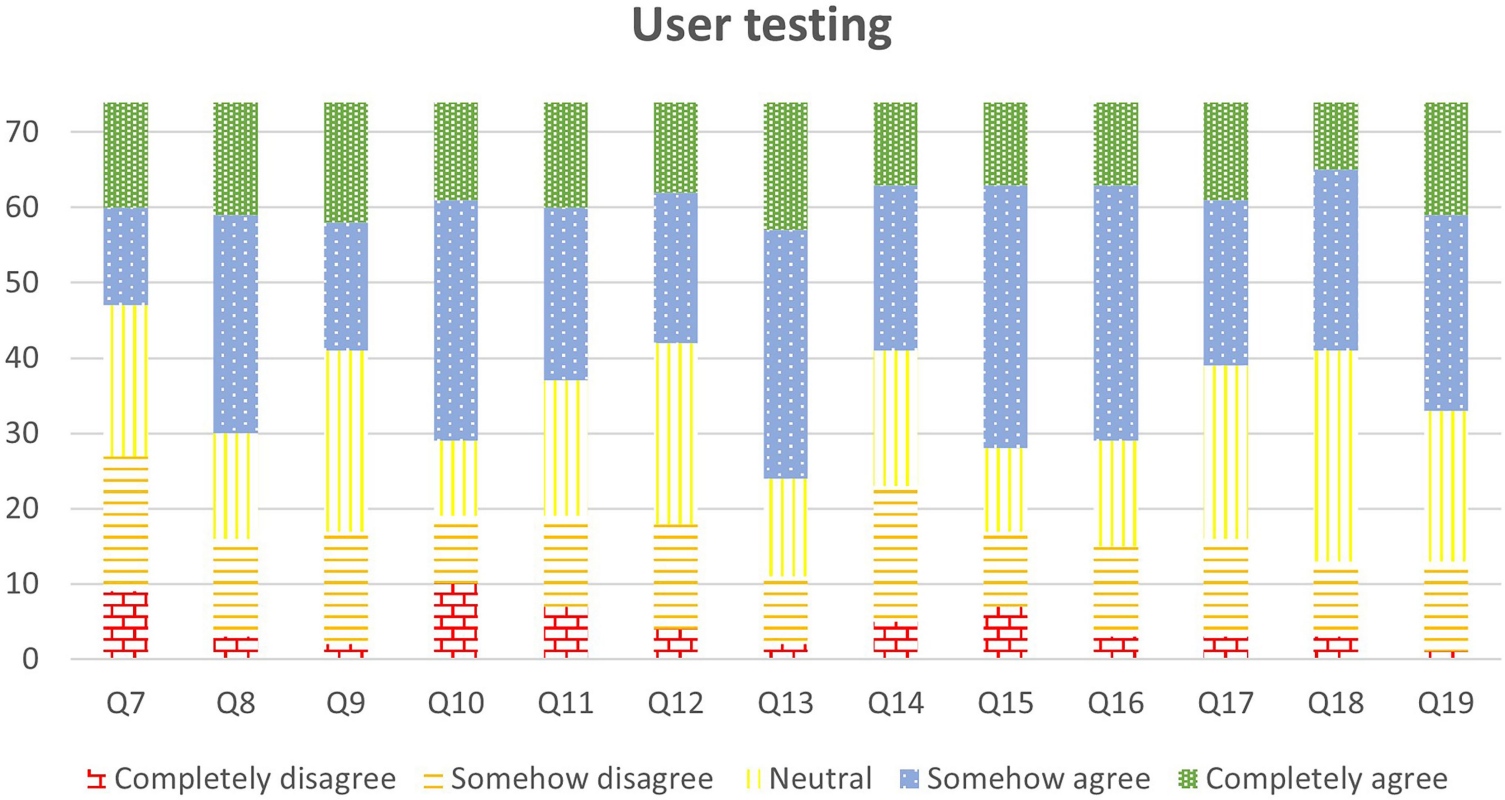
User testing results.

The quantitative evaluation for the phase 2 can be seen in Figure 13. The answers were processed in Excel and imported into SPSS Statistics 25 (License of the University of Salamanca) to conduct the statistics test. All the items are in positive, so they follow the same Likert scale.

We applied Cronbach’s alpha coefficient to check the internal consistency of each dimension. The coefficient for the sample is 0.713, which is over the recommended value of 0.7.

Question Q7–9 are related to usability. Users exposed that they knew what to do in every moment: 27 out of 74 reported agree or completely agree with the statement in Q7 (36.5%). Questions Q8 and Q9 were better valued, since users reported that they knew how to use the elements and how to move from one room to another (44–59.5%- and 33–44.6%- out of 74 responded agree, or completely agree, respectively).

With regards to the gamification strategy (Q10–Q12), 45 users reported that they, agree or completely agree with the attractiveness of the metaphor (60.8%; Q10). Q11 asks about rewards (“I obtain rewards when my performance is correct”), obtaining 37 users (50%) who reported agree or completely agree with the statement. Finally, the question related to the goal and the metaphor obtained 32 (43.2%) users who agree or completely agree with the statement (“I understand the goal I have to achieve in the metaphor”).

In the section about contents (Q13–Q16), users mostly agree that contents presented during the learning experience are proper (Q13, 50–67.6%- users agree or completely agree). They believe that the extension is not that proper (Q14, 33 out of 74 (44.6%) agree or completely agree). Quizzes are also well rated (Q15): 46 out of 74 users (62.2%) reported agreed or completely agreed. Finally, users believe that questions included in the quizzes are well linked to the content presented in the scene (Q16): 45 out of 74 users (60.8%) rated this question as agree or completely agree.

As the overall opinion, or general satisfaction, we asked learners if they liked the application (Q17), if they learned about Open Education (Q18) and if they found the information useful in their daily teaching (Q19). For Q17, 35 out of 74 (47.3%) agree or completely agree; a similar number of users reported that they had learnt about OE (33 out of 74, 44.6% agree or completely agree answers); but 41 out of 74 (55.4%) concluded that they found the application useful for their daily teaching.

Finally, we performed some addition statistical studies. In the first place, we checked if the items had a normal distribution by applying the Kolmogorov–Smirnov test. According to the results, *p* < 0.05, nonparametric tests were used to perform hypothesis contrasting.

First hypothesis contrast was focused on analyzing if there are significant differences between users from different knowledge areas. In particular, we divided answer into two groups, users from knowledge areas related to Social Science and users from Technology. We applied Mann–Whitney U because the sample is small (*N* = 74). The results show no significant differences between both groups (Table 3). We also analyzed if the academic expertise influence on the results. However, the Mann–Whitney U test does not identify significant differences among users who was teaching less than 5 years and those who have more experience (Table 3).

**Table 3:** Mann–Whitney U results for variables area and expertise (less than 5 years teaching vs. more than 5 years teaching).

	Area (*N* = 74)				Expertise (*N* = 74)		
	*U*	*Z*	Sig.		*U*	*Z*	Sig.
**Q7**	653.500	−0.207	0.836	**UQ7**	650.000	−0.361	0.718
**Q8**	626.500	−0.518	0.605	**UQ8**	642.000	−0.457	0.647
**Q9**	610.000	−0.699	0.484	**UQ9**	619.000	−0.711	0.477
**Q10**	545.000	−1.455	0.146	**GQ10**	649.500	−0.375	0.708
**Q11**	567.500	−1.174	0.241	**GQ11**	651.500	−0.345	0.730
**Q12**	660.000	−0.135	0.892	**GQ12**	577.500	−1.176	0.240
**Q13**	597.500	−0.861	0.389	**GQ13**	573.500	−1.249	0.212
**Q14**	643.000	−0.326	0.744	**CQ14**	647.500	−0.391	0.696
**Q15**	585.500	−1.004	0.316	**CQ15**	554.500	−1.474	0.141
**Q16**	653.000	−0.220	0.826	**CQ16**	565.000	−1.349	0.177
**Q17**	655.500	−0.186	0.852	**SQ17**	509.000	−1.945	0.052
**Q18**	593.500	−0.899	0.369	**SQ18**	576.000	−1.210	0.226
**Q19**	607.500	−0.732	0.464	**SQ19**	619.000	−0.715	0.475

In addition, we conducted several hypotheses contrasts to identify differences regarding a set of nominal variables: age, gender, PhD degree, and academic position. Regarding PhD degree (hold it, not hold it or be in progress) and academic position, they do not influence any of the dimensions according to the Kruskal–Wallis nonparametric test.

Regarding age, there are significant differences only in two items, Q10 and Q17 according to the Kruskal–Wallis results (see Table 4). Different results are obtained for the variable gender: it influences on items Q12 and Q18.

**Table 4:** Kruskal–Wallis results for variables age and gender.

	Age (*N* = 74)				Gender (*N* = 74)		
	*H*	gl	Sig.		*H*	gl	Sig.
**Q7**	0.184	2	0.912	**Q7**	1.514	2	0.469
**Q8**	0.488	2	0.783	**Q8**	2.870	2	0.238
**Q9**	0.299	2	0.861	**Q9**	0.236	2	0.889
**Q10**	6.188	2	**0.045**	**Q10**	2.699	2	0.259
**Q11**	0.047	2	0.977	**Q11**	4.487	2	0.106
**Q12**	1.233	2	0.540	**Q12**	6.632	2	**0.036**
**Q13**	1.683	2	0.431	**Q13**	0.175	2	0.916
**Q14**	3.143	2	0.208	**Q14**	1.368	2	0.505
**Q15**	1.702	2	0.427	**Q15**	4.996	2	0.082
**Q16**	0.367	2	0.832	**Q16**	1.189	2	0.552
**Q17**	6.517	2	**0.038**	**Q17**	1.284	2	0.526
**Q18**	0.648	2	0.723	**Q18**	6.495	2	**0.039**
**Q19**	2.144	2	0.342	**Q19**	0.970	2	0.616

Bold format for values highlighted in the discussion.

Finally, we have analyzed if understanding the goal of the game had an impact in the other items. We applied Kruskal–Wallis test for variable Q12 (I understand the goal I have to achieve in the metaphor) to conduct the hypothesis contrast. The results show significant differences in items Q7, Q10, Q13 and Q18 (Table 5).

**Table 5:** Kruskal-Wallis results for variable Q12 “I understand the goal I have to achieve in the metaphor.”

	Age (*N* = 74)		
	*H*	gl	Sig.
**Q7**	11.657	4	**0.020**
**Q8**	5.677	4	0.225
**Q9**	4.365	4	0.359
**Q10**	11.641	4	**0.020**
**Q11**	2.453	4	0.653
**Q13**	10.208	4	**0.037**
**Q14**	5.327	4	0.255
**Q15**	5.201	4	0.267
**Q16**	1.870	4	0.760
**Q17**	2.553	4	0.635
**Q18**	9.738	4	**0.045**
**Q19**	4.135	4	0.388

Bold format for values highlighted in the discussion.

## Discussion

The goal of *Catch the Open!* is engaging HE teachers in OE and OEP by using a story-based gamification approach. In this regard, questions Q13, Q15, and Q16 are the most highly rated in our survey: contents provide proper information about OE, the feedback from quizzes is clear and understandable, and questions are related to the content in the scene in which they are presented. Thus, we could say that, even if a higher proportion would be better, the goal related to instruction is sufficiently achieved.

Related to the gamification approach, most of the users think that the metaphor is attractive. Actually, it was designed to have an easy identification to our target group. Half of the users also think that rewards are obtained when they perform correctly, but some more clear information about that could be helpful in this regard. Unfortunately, a few less that the half of users understood the goal of the metaphor. From the open opinion field provided in the questionnaire, we can assume that a ‘Back button” was missed by some of the users, since they explained that they would have liked to read some of the dialogues again. In addition, we could guess that the low rating in questions about usability (Q7 -I know what to do in each moment- is the lowest rated question) are also related to that point, since dialogues give the clues to understand what to do, what to use and how to use different elements in the scenario. If users miss the information in dialogues, they can feel lost and not to know how to proceed.

Moreover, we find significant differences when we analyze the question Q12 (“I understand the goal I have to achieve in the metaphor”). Those differences impact on the users’ opinion about what they have to do in each moment (Q7), their perception about the attractiveness of the metaphor (Q10), the properness of the content (Q13), and how many they learnt about OE (Q18). This can be explained underpinned by several theories already exposed in this paper: the playful experience is needed to enjoy and be engaged in the learning process (Wang et al., 2021). If users do not understand the metaphor properly, they are not able to or interested in following the story, losing information to advance in it and, consequently, to access to the contents. Moreover, we find that this is particularly visible if we observe the polarization of profiles in these questions: they are better rated when they are answered by non-feminine users, doctors or PhD students, or novel users in teaching, or non-technological-profiled users.

### Lessons Learned

When the partnership worked in the gamified experience design, we tried to get the application close to a real experience for players. However, after several rounds of evaluation and refinement of the application, we learned some lessons to be taken into consideration for next projects:

Metaphor: An understandable metaphor helps users to get identified with the narrative and enhance the immersive experience.

Configuration: Since the target group can include a wide range of ages, experience, and backgrounds, the experience should be guided or, at least, offer some settings to configure what level of help the user needs. In this regard, users in the evaluation described in this paper indicated the need of a “back” button to fully understand instructions and, that way, understand what to do in each of the rooms. This way, users not used to playing game can feel more comfortable with the interface working.

Feedback: Users need to know they obtain a reward. For example, some sounds or blinking markup to make him or her aware of the advance.

At-the-point contents: Too long contents make users spend too much time, considering our target group. Thus, we need to limit the quantity of hours to complete the learning process.

## Conclusion and Future Research Directions

This paper presents the main outcome of the OpenGame project, which is the gamified web-based application *Catch the Open!* This application offers a gamified course based on theoretical foundations and concepts of OE together with a set of real-life open education practices, which give the user the opportunity to get inspired to start or to innovate in the use of open educational practices (OEP). Moreover, this content is available as OER (de la Higuera et al., 2020) and was developed to overcome the competences framework (Garcia-Holgado et al., 2020) for OE developed in the OpenGame project.

*Catch the Open!* was developed following a user-centered approach, around a well-known metaphor for the target group: the usual activities of an enthusiastic HE educator who tries to help her university to get involved in OE. In order to obtain a good solution, several partial tests were performed, both internally (in the consortium) and with potential users. After two rounds of tests, the final version of the application was launched in September 2021.

From the heuristic assessment, we found several elements to be improved related to *game mechanics* in order to ease users to feel free in the game universe. Unfortunately, some of those problems remained in view of the usability ratings. However, as follows from the statistical analysis, if users understand properly the metaphor, that usability problems decrease, since paying attention to the story helps to understand some of the clues to advance in the experience.

On the other hand, we can say that the application *Catch the open!* represents a helpful tool for teacher. The information about EP and OEP was properly rated by users and the general satisfaction with the web application was mostly rated to be useful for its purpose, in a sample of 74 users.

After the study presented in this paper, underpinned by a great dissemination activity, the application achieved a number of 255 users and that number is intended to increase by the use of *Catch the Open!* in several universities by current and future HE educators, giving the opportunity to study what modules are the most and the less frequently visited, as well as provide information when requested, in order to improve the experience.

## Data Availability Statement

The raw data supporting the conclusions of this article will be made available by the authors, without undue reservation.

## Ethics Statement

Ethical review and approval was not required for the study on human participants in accordance with the local legislation and institutional requirements. The patients/participants provided their written informed consent to participate in this study.

## Author Contributions

NP-Z and DB led the writing process as well as sections “Related Works” and “Research Method.” AG-H and FG-P led the sections “OE and Formative Opportunities for HE Educators: The Current Situation,” “*Catch the Open!* The Technological Approach”, and as well as the statistical analysis. Cd-l-H and MH led section “Related works”. JB led section “Introduction” and developed a critical review of the paper. AT led section “Results” and developed a critical review of the paper. All authors contributed to the article and approved the submitted version.

## Funding

This research was partially supported by the project OpenGame (2019-1-ES01-KA203-065815) funded by the European Commission under the Erasmus+ Programme and the Research Institute for Innovation & Technology in Education (UNIR iTED) at the Universidad Internacional de La Rioja (UNIR).

## Conflict of Interest

The authors declare that the research was conducted in the absence of any commercial or financial relationships that could be construed as a potential conflict of interest.

## Publisher’s Note

All claims expressed in this article are solely those of the authors and do not necessarily represent those of their affiliated organizations, or those of the publisher, the editors and the reviewers. Any product that may be evaluated in this article, or claim that may be made by its manufacturer, is not guaranteed or endorsed by the publisher.

## Footnotes

^1^ https://library.shout4he.eu/

^2^ https://exploerer.wordpress.com/

^3^ https://openmedproject.eu/

^4^ https://encoreproject.eu/
